# Off-Label Antipsychotic Prescribing for Persons With Dementia in Assisted Living

**DOI:** 10.1093/geroni/igaa057.2483

**Published:** 2020-12-16

**Authors:** Christopher Wretman, Sheryl Zimmerman, Philip Sloane, John Preisser

**Affiliations:** University of North Carolina at Chapel Hill, Chapel Hill, North Carolina, United States

## Abstract

Off-label antipsychotic use is contra-indicated for persons with dementia due to lack of efficacy and FDA black box warnings. Yet, residents in assisted living (AL), including those with dementia, are frequently prescribed such medications. If distinct typologies of AL communities exist based on prescribing rates, it might be possible to reduce use. Toward that end, data from 247 AL communities in seven states were analyzed to discern potential unobserved latent classes that differentiate prescribing levels. Results using finite mixture modeling determined a 5-class solution best fit the data, with class-level prescribing rates ranging from 16.9% of residents with dementia to 27.4% (Mean = 18.9%). Bivariate tests found differences across classes by variables related to community structure, medication processes, and resident-case mix (e.g., frequent formal pharmacy review was more likely in communities with higher prescribing). Typologies are useful to identify differences and care and may be useful for quality improvement.

